# Air pollution and mortality in a large, representative U.S. cohort: multiple-pollutant analyses, and spatial and temporal decompositions

**DOI:** 10.1186/s12940-019-0544-9

**Published:** 2019-11-21

**Authors:** Jacob S. Lefler, Joshua D. Higbee, Richard T. Burnett, Majid Ezzati, Nathan C. Coleman, Dalton D. Mann, Julian D. Marshall, Matthew Bechle, Yuzhou Wang, Allen L. Robinson, C. Arden Pope

**Affiliations:** 1 Department of Agricultural and Resource Economics, University of California, Berkeley, CA 94720 USA; 2 Department of Economics, University of Chicago, Chicago, IL USA; 3 Health Canada, Ottawa, Ontario Canada; 4 MRC Centre for Environment and Health, School of Public Health, Imperial College London, London, UK; 5 Department of Economics, Brigham Young University, Provo, UT USA; 6 Department of Civil and Environmental Engineering, University of Washington, Seattle, WA USA; 7 Engineering and Public Policy, Carnegie Mellon University, Pittsburgh, PA USA

**Keywords:** Air pollution, Particulate matter, Sulfur dioxide, Mortality, Cardiopulmonary disease

## Abstract

**Background:**

Cohort studies have documented associations between fine particulate matter air pollution (PM_2.5_) and mortality risk. However, there remains uncertainty regarding the contribution of co-pollutants and the stability of pollution-mortality associations in models that include multiple air pollutants. Furthermore, it is unclear whether the PM_2.5_-mortality relationship varies spatially, when exposures are decomposed according to scale of spatial variability, or temporally, when effect estimates are allowed to change between years.

**Methods:**

A cohort of 635,539 individuals was compiled using public National Health Interview Survey (NHIS) data from 1987 to 2014 and linked with mortality follow-up through 2015. Modelled air pollution exposure estimates for PM_2.5_, other criteria air pollutants, and spatial decompositions (< 1 km, 1–10 km, 10–100 km, > 100 km) of PM_2.5_ were assigned at the census-tract level. The NHIS samples were also divided into yearly cohorts for temporally-decomposed analyses. Cox proportional hazards models were used to estimate hazard ratios (HRs) and 95% confidence intervals (CIs) in regression models that included up to six criteria pollutants; four spatial decompositions of PM_2.5_; and two- and five-year lagged mean PM_2.5_ exposures in the temporally-decomposed cohorts. Meta-analytic fixed-effect estimates were calculated using results from temporally-decomposed analyses and compared with time-independent results using 17- and 28-year exposure windows.

**Results:**

In multiple-pollutant analyses, PM_2.5_ demonstrated the most robust pollutant-mortality association. Coarse fraction particulate matter (PM_2.5–10_) and sulfur dioxide (SO_2_) were also associated with excess mortality risk. The PM_2.5_-mortality association was observed across all four spatial scales of PM_2.5_, with higher but less precisely estimated HRs observed for local (< 1 km) and neighborhood (1–10 km) variations. In temporally-decomposed analyses, the PM_2.5_-mortality HRs were stable across yearly cohorts. The meta-analytic HR using two-year lagged PM_2.5_ equaled 1.10 (95% CI 1.07, 1.13) per 10 μg/m^3^. Comparable results were observed in time-independent analyses using a 17-year (HR 1.13, CI 1.09, 1.16) or 28-year (HR 1.09, CI 1.07, 1.12) exposure window.

**Conclusions:**

Long-term exposures to PM_2.5_, PM_2.5–10_, and SO_2_ were associated with increased risk of all-cause and cardiopulmonary mortality. Each spatial decomposition of PM_2.5_ was associated with mortality risk, and PM_2.5_-mortality associations were consistent over time.

## Background

Numerous studies have documented associations between long-term exposure to fine particulate matter air pollution (PM_2.5_, particles < 2.5 μm in aerodynamic diameter) and risk of mortality. Notable cohort studies have indicated that elevated PM_2.5_ exposures are associated with increased risks of all-cause and cardiopulmonary mortality [1–25]. Several studies have estimated the association between PM_2.5_ and mortality while controlling for exposures to one or more co-pollutants, such as ozone (O_3_), nitrogen dioxide (NO_2_), and sulfur dioxide (SO_2_) [4, 5, 13, 20]. There remains a need for further multiple-pollutant analyses that control for other common air pollutants, including coarse fraction particulate matter (PM_2.5–10_, particles 2.5–10 μm in aerodynamic diameter) and carbon monoxide (CO).

Related to multiple-pollutant analyses are models that examine constituents of PM_2.5_ rather than aggregated PM_2.5_ treated as a single pollutant. The composition and toxicity of PM_2.5_ can vary substantially based on when and where it is sampled and the distance from the pollution source [26, 27]. Exposures that occur near a pollution source may include a larger fraction of primary combustion products (black carbon and primary organic aerosol) and other local sources (industrial and road dust). Alternatively, exposure may occur farther from the source, allowing a larger fraction of aged, agglomerated, and secondary particulate matter (sulfates, nitrates, and secondary organic aerosol). Are there differences in the PM_2.5_-mortality associations across spatial decompositions of PM_2.5_ pollution?

The composition of PM_2.5_ not only varies spatially, but may also vary temporally as sources of pollution change. Furthermore, ambient pollution levels change over time, and observed health effects of PM_2.5_ likely depend on the window of exposure assigned to individuals in the cohort. Therefore, an important question is, are there differences in observed PM_2.5_-mortality associations across time or for different windows of PM_2.5_ exposure?

This study uses a large, well-documented, and representative cohort of the U.S. [25] to pursue three primary objectives. First, investigate pollution-mortality associations with models that include multiple air pollutants. Second, explore differences in PM_2.5_-mortality associations across spatially-decomposed PM_2.5_ as an evaluation of whether the impact of PM_2.5_ depends on distance from pollution source. Third, estimate PM_2.5_-mortality associations in temporally-decomposed cohorts, allowing effect estimates to vary across time and for different choices of exposure window.

## Methods

### Study population

The cohort for this study was constructed using publicly-available National Health Interview Survey (NHIS) data from 1987 to 2014, linked with restricted-use geographic information and mortality follow-up through 2015. The sample was limited to NHIS respondents aged 18–84 at the time of survey for whom information was available regarding age, sex, race-ethnicity, income, education, marital status, smoking status, BMI, census tract, ambient air pollution, survey date, mortality status at the end of 2015, and date of death (if deceased at the end of 2015).

The NHIS is a household survey administered annually by the National Center for Health Statistics (NCHS) and designed to be representative of the civilian noninstitutionalized U.S. population [28]. Survey data were linked with the National Death Index for mortality follow-up through 2015 [29]. The construction of this cohort has been described in a previous study [25], where it was referred to as a “subcohort” of a larger NHIS cohort. This cohort, rather than the larger “full cohort” of the prior study, was chosen for the present analysis because it included information for smoking status and BMI. The NHIS design was altered periodically over the sample period, so some variables required harmonization. Data linkage was performed with permission and assistance from the NCHS. Further details on construction, harmonization, and data linkage for the NHIS cohort are documented elsewhere [25].

### Air pollution data

Air pollution exposures were assigned to individuals based on their census tract of residence at the time of survey, using year-2000 Census tracts for individuals surveyed from 1987 to 2010 and year-2010 Census tracts for individuals surveyed from 2011 to 2014. Annual-average estimates of ambient air pollution were calculated for criteria pollutants (PM_2.5_, PM_10_, SO_2_, NO_2_, O_3_, and CO) using estimates from the v1 empirical models of Kim et al., 2018 [30], available at www.caces.us. These models employed regulatory monitoring and land-use data, and pollution estimates were calculated starting with the first year for which nationwide monitoring data were available for that pollutant (1979 for SO_2_, NO_2_, and O_3_; 1988 for PM_10_; 1990 for CO; and 1999 for PM_2.5_). In the case of O_3_, annual values are the mean for May through September of the daily maximum eight-hour moving average. O_3_ monitoring is not widely and routinely conducted from October through April since these months typically experience very low O_3_ concentrations. Estimates for each pollutant-year through 2015 were generated at the census-block level using year-2010 Census block centroids. Tract-level estimates for year-2000 Census tracts and year-2010 Census tracts were estimated by mapping year-2010 Census blocks to census tracts and then calculating a population-weighted average of the census blocks within a census tract. PM_2.5_ exposures prior to 1999 were estimated by multiplying a census tract’s PM_10_ value with the census tract’s mean PM_2.5_:PM_10_ ratio from 1999 to 2003, as explained elsewhere [25]. Values for PM_2.5–10_ were calculated by subtracting PM_2.5_ from PM_10_.

In addition, spatially-decomposed PM_2.5_ data were generated following an approach described elsewhere [26]. Briefly, a census block’s total ambient PM_2.5_ was decomposed into four components, depending on the spatial variance in PM_2.5_ surrounding the census block. Estimating spatial decompositions involved finding and subtracting the minimum PM_2.5_ values within circular buffers around each census block. First, the minimum PM_2.5_ for census block centroids within a 100 km radius of a given census block centroid was found, and this minimum was designated as regional (> 100 km) PM_2.5_. After subtracting regional PM_2.5_, the minimum PM_2.5_ within a 10 km radius of the census block centroid was found, and this value was designated as mid-range (10–100 km) PM_2.5_. Next, the minimum value within 1 km of the block centroid was similarly used to calculate neighborhood (1–10 km) PM_2.5_ by subtracting regional and mid-range PM_2.5_. Finally, the residual PM_2.5_ that remained after subtracting regional, mid-range, and neighborhood PM_2.5_ was called local (< 1 km) PM_2.5_. The process was repeated for each year-2010 Census block and for each year from 2000 through 2015. Values for census tracts were calculated using population-weighted averages of year-2010 Census blocks.

### Statistical analyses

Statistical analyses were performed at the NCHS Research Data Center in Hyattsville, MD, using SAS (version 9.3; SAS Institute). Survival analyses were performed for all-cause and cardiopulmonary mortality, with cardiopulmonary mortality defined as mortality due to cardiovascular disease (ICD-10 codes: I00-I09, I11, I13, I20-I51), cerebrovascular disease (I60-I69), chronic lower respiratory disease (J40-J47), and influenza or pneumonia (J09-J18). Mortality hazard ratios (HRs) and 95% confidence intervals (CIs) were estimated using two versions of the Cox proportional hazards (PH) model. The first PH model, referred to as the basic PH model, controlled for age, sex, and race-ethnicity by allowing each combination of age (in one-year increments), sex, and race-ethnicity (Hispanic, non-Hispanic black, non-Hispanic white, other or unknown) its own baseline hazard function using the STRATA statement of the PHREG procedure in SAS. The second PH model, referred to as the complex PH model, controlled for age group (18–24 years and subsequent five-year age groups), sex, and race-ethnicity by including an indicator variable for each interaction of age group, sex, and race-ethnicity. The complex PH model was estimated using the SURVEYPHREG procedure in SAS, adjusting for the NHIS complex survey design, using reported survey stratum, primary sampling unit, and sample weight from mortality follow-up files [28].

Both PH models controlled for covariates by including indicator variables for each value of marital status (never married, married, separated, divorced, widowed), inflation-adjusted household income ($0–35,000; $35,000-50,000; $50,000-75,000; >$75,000), education (<high school graduate, high school graduate, some college, college graduate, >college graduate), smoking status (current, former, never), BMI (< 20, 20–25, 25–30, 30–35, > 35), U.S. Census region, urban versus rural designation, and survey year. Survival time was the number of days between survey and death. For all-cause mortality, censored survival time was the number of days between survey and mortality follow-up (31 Dec 2015). In models that considered cardiopulmonary mortality, censored survival time was the number of days between survey and mortality follow-up, or the number of days between survey and non-cardiopulmonary mortality. Pollution values were included as continuous variables in the regressions.

In models using criteria pollutants (PM_2.5_, PM_2.5–10_, SO_2_, NO_2_, O_3_, and CO), regressions included one, two, or six pollutants, and were estimated for both all-cause and cardiopulmonary mortality. One- and two-pollutant regression models used the basic PH model. For six-pollutant regression models, both the basic and complex PH models were employed to examine whether results were sensitive to adjusting for the NHIS complex survey design. Basic PH models were also used to estimate the associations between spatial decompositions of PM_2.5_ and risk of all-cause and cardiopulmonary mortality. Regressions were performed for each of the four decompositions individually and for models that included all four decompositions.

For temporally-decomposed analyses, the NHIS cohort was decomposed into 24 yearly cohorts (1992–2015), beginning in 1992 to allow up to a five-year lagged pollution-exposure window. An individual in the NHIS cohort was included in a particular year’s cohort if she was alive on 1 Jan and was surveyed by 31 Dec of that year. For example, the 1992 cohort included those surveyed before 1992 and alive on 1 Jan 1992. It also included those who were surveyed in 1992. For those who died in 1992, survival time was the number of days between 1 Jan 1992 and date of death (for individuals surveyed before 1992), or the number of days between survey date and date of death (for individuals surveyed in 1992). For those who did not die in 1992, censored survival time was the number of days between 1 Jan and 31 Dec (for individuals surveyed before 1992), or the number of days between survey date and 31 Dec (for individuals surveyed in 1992). Analogous cohorts were constructed for each year from 1993 to 2015. The construction of these yearly cohorts is illustrated in Additional file 1: Figure S1.

Complex PH regressions were performed for all-cause and cardiopulmonary mortality for each of the 24 temporally-decomposed cohorts. In each cohort, individuals were assigned a two-year (cohort year and previous year) and five-year (cohort year and four previous years) average of ambient PM_2.5_ using their census tract of residence at time of survey. In addition, age was adjusted to age in cohort year. Other covariates were not updated between cohorts. Meta-analytic fixed-effect estimates of the HR associated with a 10 μg/m^3^ increase in mean ambient PM_2.5_ were calculated for all-cause and cardiopulmonary mortality using estimates generated by the 24 yearly cohorts (Comprehensive Meta Analysis Ver. 3 Biostat Englewood, NJ).

## Results

**Fig. 1:**
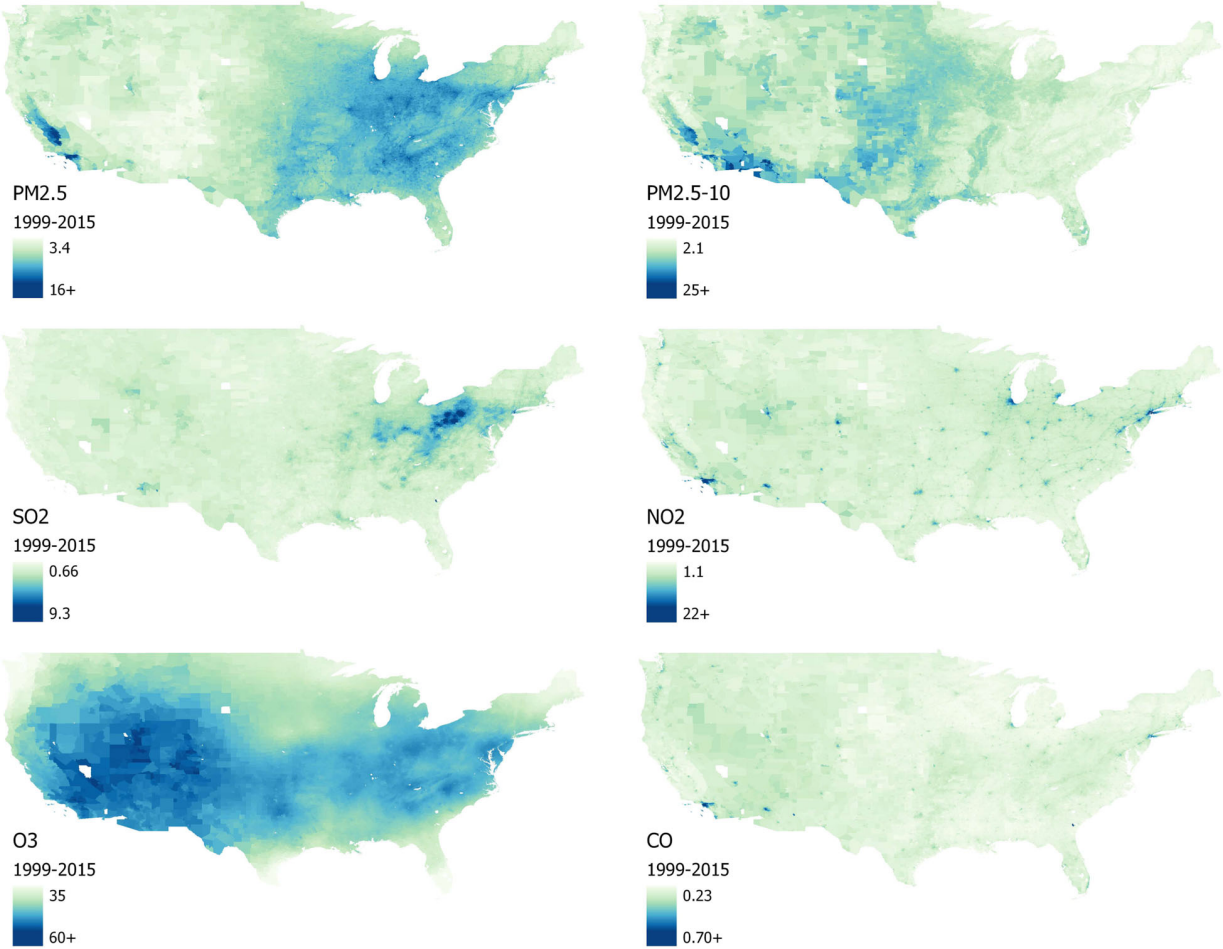
Average concentrations of criteria pollutants by 2010 Census tracts in the continental U.S., 1999–2015. PM_2.5_, fine particulate matter (particles < 2.5 μm in aerodynamic diameter) in μg/m^3^; PM_2.5–10_, coarse fraction particulate matter (particles 2.5–10 μm in aerodynamic diameter) in μg/m^3^; SO_2_, sulfur dioxide in ppb; NO_2_, nitrogen dioxide in ppb; O_3_, ozone in ppb, mean for May–September of daily max of eight-hour moving average; CO, carbon monoxide in ppm

Table 1 presents summary statistics for the NHIS cohort. Table 2 provides summary statistics (mean, standard deviation, and interquartile range [IQR]) for the 17-year (1999–2015) averages of the six criteria pollutants (PM_2.5_, PM_2.5–10_, SO_2_, NO_2_, O_3_, and CO) and correlation coefficients between pollutants, within the NHIS cohort. Criteria pollutants were generally positively correlated, with the exception of PM_2.5–10_ and SO_2_ (see Table 2). Figure 1 presents heat maps for the six criteria pollutants across census tracts in the contiguous U.S.

**Table 1:** Baseline unweighted characteristics of the NHIS cohort

Variable	NHIS Cohort
Total number in cohort	635,539
Total Deaths	106,385
Cardiopulmonary^a^	43,195
Sex	
% Male	44.54
% Female	55.46
Age yrs. (mean)	45.3
Race/Ethnicity	
% Non-Hispanic White	67.51
% Hispanic	14.08
% Non-Hispanic Black	14.01
% All other/unknown	4.40
Income (inflation adjusted to 2015)	
% $ 0–35,000	38.04
% $ 35–50,000	15.47
% $ 50–75,000	18.79
% $ 75,000+	27.71
Marital Status	
% Married	49.57
% Divorced	14.06
% Separated	3.59
% Never Married	24.31
% Widowed	8.47
Education	
% < High School grad	18.63
% High School grad	30.37
% Some College	27.10
% College grad	15.03
% > College grad	8.87
Urban/Rural	
% Urban	77.64
% Rural	22.36
Census Region	
% Northeast	18.08
% Midwest	23.71
% South	35.74
% West	22.46
BMI	
% < 20	7.28
% 20–25	36.37
% 25–30	33.80
% 30–35	14.43
% > 35	8.12
Smoking	
% Never	53.76
% Current	23.90
% Former	22.34

^a^Cardiopulmonary mortality is based on International Statistical Classification of Diseases, Injuries, and Causes of Death, Tenth Revision (ICD-10) and includes: cardiovascular disease (I00-I09, I11, I13, I20-I51), cerebrovascular disease (I60-I69), chronic lower respiratory disease (J40-J47), and influenza and pneumonia (J09-J18)

**Table 2:** Correlations (Pearson’s r) and summary statistics of criteria pollutants (1999–2015) in the NHIS cohort

	PM_2.5_	PM_2.5–10_	SO_2_	NO_2_	O_3_	CO	Mean	SD	IQR
PM_2.5_ (10 μg/m^3^)							10.67	2.37	3.12
PM_2.5–10_ (10 μg/m^3^)	0.19						9.77	4.51	5.42
SO_2_ (ppb)	0.41	−0.34					2.25	1.01	1.26
NO_2_ (ppb)	0.56	0.44	0.37				10.69	5.73	6.72
O_3_ (ppb)	0.33	0.17	0.17	0.11			47.45	5.31	6.75
CO (ppm)	0.42	0.56	0.17	0.90	0.04		0.37	0.10	0.10

Note: PM_2.5_, fine particulate matter (particles < 2.5 μm in aerodynamic diameter); PM_2.5–10_, coarse fraction particulate matter (particles 2.5–10 μm in aerodynamic diameter); SO_2_, sulfur dioxide; NO_2_, nitrogen dioxide; O_3_, ozone, mean for May–September of daily max of eight-hour moving average; CO, carbon monoxide; SD, standard deviation; IQR, interquartile range

**Fig. 2:**
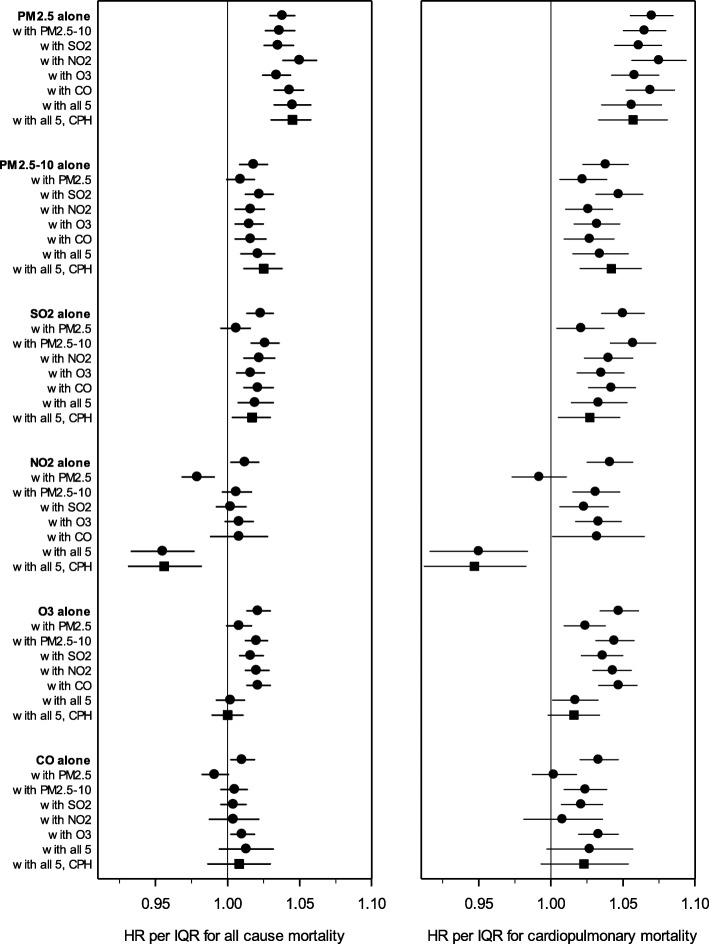
Illustration of regression results using 6 criteria pollutants, examining all-cause (left panel) and cardiopulmonary (right panel) mortality. Hazard ratios (and 95% CIs) were estimated using models that adjusted for age, sex, race-ethnicity, marital status, inflation-adjusted household income, education, smoking status, BMI, U.S. Census region, urban versus rural designation, and survey year. Hazard ratios are represented with circles when estimated using basic proportional hazards regressions, and with squares when estimated using complex proportional hazards (PH) regressions. Data used to generate plot are listed in Additional file 1 Table S1.

Figure 2 illustrates the HRs (and 95% CIs) estimated in regression models with the six criteria pollutants, using one-, two-, and six-pollutant models. HRs and CIs in Fig. 2 are presented relative to each pollutant’s IQR. Exposure to PM_2.5_ was consistently associated with increased risk of all-cause and cardiopulmonary mortality, and the PM_2.5_-mortality associations were statistically significant and insensitive to controlling for other pollutants. Exposures to PM_2.5–10_ and SO_2_ were also associated with increased mortality risk, including in six-pollutant models, but the associations were less robust. NO_2_, O_3_, and CO were not consistently linked with excess mortality risk. In models that controlled for PM_2.5_, exposures to NO_2_ were associated with reduced mortality risk. Furthermore, O_3_ was not associated with excess risk of all-cause mortality in six-pollutant models, and O_3_-mortality associations were marginally significant in six-pollutant cardiopulmonary regression models. Estimated HRs were not sensitive to using the complex PH regression model.

Because the IQR of PM_2.5–10_ (5.42 μg/m^3^) is larger than the IQR of PM_2.5_ (3.12 μg/m^3^), the pollution-mortality HRs associated with these two pollutants appear more similar in Fig. 2 than when scaled by 10 μg/m^3^. In the two-pollutant basic PH model with PM_2.5_ and PM_2.5–10_, the all-cause mortality HR associated with a 10 μg/m^3^ increase in PM_2.5_ is 1.12 (95% CI: 1.09, 1.15), whereas the HR associated with a 10 μg/m^3^ increase in PM_2.5–10_ is 1.02 (1.00, 1.04). Thus, when considered per 10 μg/m^3^, exposure to PM_2.5_ is associated with about six times greater excess risk than PM_2.5–10_.

Table 3 provides summary statistics and correlations for 16-year (2000–2015) averages of spatial decompositions of PM_2.5_ (local PM_2.5_, < 1 km; neighborhood PM_2.5_, 1–10 km; mid-range PM_2.5_, 10–100 km; regional PM_2.5_, > 100 km), within the NHIS cohort. Although local, neighborhood, and mid-range PM_2.5_ are somewhat correlated, regional PM_2.5_ is mostly uncorrelated with local PM_2.5_ and negatively correlated with neighborhood and mid-range PM_2.5_ (see Table 3). Table 3 reports large differences in the means and IQRs of the spatial decompositions of PM_2.5_.

**Table 3:** Correlations (Pearson’s r) and summary statistics for spatial decompositions of PM_2.5_ (2000–2015) in the NHIS cohort

	Local (< 1 km)	Neighborhood (1–10 km)	Mid-range (10–100 km)	Regional (> 100 km)	Mean	SD	IQR
Local PM_2.5_					0.63	0.28	0.32
Neighborhood PM_2.5_	0.25				1.81	0.87	1.01
Mid-range PM_2.5_	0.17	0.29			2.59	1.45	1.53
Regional PM_2.5_	0.01	−0.33	−0.21		5.47	1.90	2.65

Note: Local PM_2.5_, PM_2.5_ generated within 1 km of residence; neighborhood PM_2.5_, PM_2.5_ generated 1–10 km from residence; mid-range PM_2.5_, PM_2.5_ generated 10–100 km from residence; regional PM_2.5_, PM_2.5_ generated over 100 km from residence; SD, standard deviation; IQR, interquartile range

**Fig. 3:**
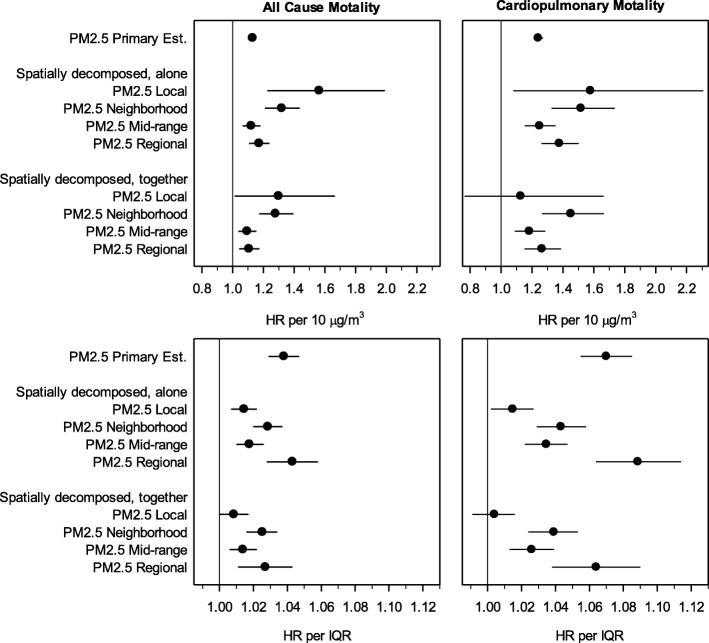
Illustration of spatially-decomposed analyses, presented per 10 μg/m^3^ (top panel) and per IQR (bottom panel). Hazard ratios (and 95% CIs) were estimated using the basic proportional hazards regressions model which adjusted for age, sex, race-ethnicity, marital status, inflation-adjusted household income, education, smoking status, BMI, U.S. Census region, urban versus rural designation, and survey year. Local PM_2.5_, PM_2.5_ generated within 1 km of residence; neighborhood PM_2.5_, PM_2.5_ generated 1–10 km from residence; mid-range PM_2.5_, PM_2.5_ generated 10–100 km from residence; regional PM_2.5_, PM_2.5_ generated over 100 km from residence; IQR, interquartile range. Data used to generate plot are listed in Additional file 1 Table S2.

Fig. 3 presents estimated HRs for all-cause and cardiopulmonary mortality from models including spatially-decomposed PM_2.5_. In the top panel, HRs are presented per 10 μg/m^3^ to assess the toxicity of spatial components of particulate matter. The same results are also presented as scaled by IQR (bottom panel) to account for differences in exposure variability across spatial decompositions of PM_2.5_. Regression results from models that included individual spatial decompositions were comparable to results from models that included all four spatial decompositions. Both types of model provide some evidence that local PM_2.5_ and neighborhood PM_2.5_ may be more toxic than mid-range and regional PM_2.5_.

**Fig. 4:**
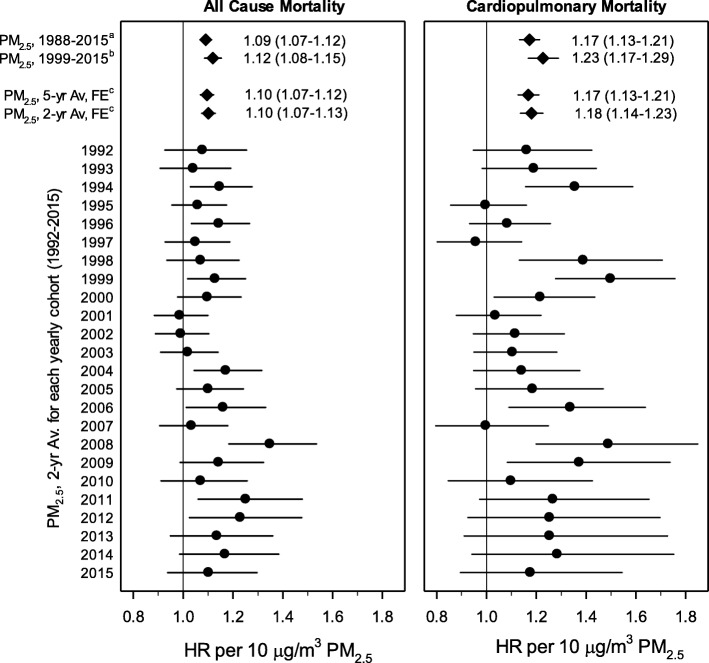
Illustration of temporally-decomposed and related analyses. Hazard ratios (and 95% CIs) for temporally-decomposed cohort analyses estimated using the complex proportional hazards regression model adjusting for age, sex, race-ethnicity, marital status, inflation-adjusted household income, education, smoking status, BMI, U.S. Census region, urban versus rural designation, and survey year. Cardiopulmonary mortality is based on ICD-10 codes and includes: cardiovascular disease (I00-I09, I11, I13, I20-I51), cerebrovascular disease (I60-I69), chronic lower respiratory disease (J40-J47), and influenza and pneumonia (J09-J18). Data used to generate plot are listed in Additional file 1 Table S3. ^a^Time-independent estimate using 17-yr (1999–2015) mean PM_2.5_ in the complex proportional hazards regression model [25]. ^b^Time-independent estimate using 28-yr (1988–2015) mean PM_2.5_ in the basic proportional hazards regression model, with back-casted PM_2.5_ data for 1988 through 1998 [25]. ^c^Cohort results using two- and five-year lagged PM_2.5_ were pooled using fixed-effect (FE) meta-analysis.

Fig. 4 presents results from the temporally-decomposed analysis. HRs for all-cause and cardiopulmonary mortality associated with a 10 μg/m^3^ increase in two-year mean PM_2.5_ are presented from regressions performed on the 24 temporally-decomposed cohorts. These PM_2.5_-mortality associations were consistent across follow-up years. Although PM_2.5_-mortality associations were generally not statistically significant for individual cohort years, meta-analytic estimates of pooled results were statistically significant. HRs from fixed-effect meta-analyses of HRs from the 24 cohorts are also presented for two- and five-year mean PM_2.5_ and for all-cause and cardiopulmonary mortality. HRs associated with two-year and five-year mean PM_2.5_ were nearly identical. Also presented are HRs from time-independent analyses which used the entire NHIS cohort and 17-year (1999–2015) or 28-year (1988–2015) mean PM_2.5_. HRs from meta-analyses of temporally-decomposed regressions were greater than HRs associated with 28-year mean PM_2.5_ but less than HRs associated with 17-year mean PM_2.5_.

## Discussion

This study advances our understanding of mortality risk associated with long-term exposure to PM_2.5_ in several ways. First, it illustrates that the PM_2.5_-mortality association within a large cohort is not highly sensitive to controlling for other air pollutants. Second, results from multiple-pollutant models report that, while mortality risk associated with PM_2.5_ exposure was the most prominent and robust result, exposures to elevated levels of SO_2_ and PM_2.5–10_ were also consistently linked to excess mortality risk. Third, regressions using spatially-decomposed PM_2.5_ suggest that more spatially variable components (< 10 km) of PM_2.5_ exposures may be more toxic. Fourth, mortality risk was significantly associated with all spatial decompositions of PM_2.5_, indicating that the PM_2.5_-mortality association within the U.S. is likely not the result of exclusively regional or local confounders. And fifth, the temporally-decomposed analysis indicates that PM_2.5_-mortality associations were largely consistent over time within the NHIS cohort, but provides incomplete evidence regarding the most relevant window of pollution exposure.

The robustness of the PM_2.5_-mortality association has been reported by various studies, including studies using two- or three-pollutant models [4, 5, 13, 20]. Our results regarding risks associated with other air pollutants, however, were less congruent with existing literature. For example, this study found a relatively stable association between PM_2.5–10_ and mortality, which contrasts with the lack of consistent associations in similar cohort studies [31]. Similarly, previous studies examining the effect of long-term O_3_ exposures reported results that remained significant when controlling for PM_2.5_ and NO_2_ [4, 13, 20], while this study found that the association was stable except in six-pollutant models. The mortality association with NO_2_ was extremely sensitive to the inclusion of other pollutants, especially PM_2.5_. Ultimately, the clearest signals emerging from multiple-pollutant regressions were that the PM_2.5_-mortality association was the most robust among these pollutants and that the mortality associations of other pollutants require further investigation.

The spatially-decomposed analyses are interesting because they provide insight into different components of PM_2.5_. PM_2.5_ is largely comprised of regional and mid-range components which are presumably dominated by secondary material (sulfates, nitrates, and secondary organic aerosol). The neighborhood and local components contribute a relatively small fraction of the PM_2.5_ mass (6 and 17% respectively) but are presumably more influenced by local emissions and therefore comprised of combustion emissions (black carbon and primary organic aerosol) and other local sources (industrial and road dust). As illustrated in Fig. 3, these results provide some evidence that local PM_2.5_ and neighborhood PM_2.5_ may be more strongly associated with mortality risk than regional PM_2.5_. Near-source PM_2.5_ was also more strongly associated with mortality risk than regional PM_2.5_ in another large U.S. cohort [32]. An implication of these results is that reliance on PM_2.5_-mortaltiy associations that are driven largely by regional differences in pollution may underestimate the health effects of exposure to local sources of pollution.

Strengths of the NHIS cohort have been described previously [25], which include the availability of detailed documentation, precise geographic information, large sample size, representativeness of U.S. adults, and individual-level controls for age, race-ethnicity, sex, smoking status, education, BMI, marital status, and income. Other strengths of this study include *a*) the robustness of the PM_2.5_-mortality association in multiple-pollutant models that included modeled air pollution estimates for six criteria pollutants. *b*) The ability to examine the stability of other pollutant-mortality associations in multiple-pollutant models. *c*) The use of spatially-decomposed PM_2.5_ data to investigate whether the toxicity of PM_2.5_ depended on proximity to source. *d*) Temporally-decomposed analyses which allowed exposures and mortality effects to vary between years and facilitated comparisons of different windows of exposure.

This study also has important limitations. Like all observational studies, it was hindered by a lack of random exposure assignment, meaning it was susceptible to confounders that were unobserved or inadequately controlled for. Another limitation was the lack of follow-up for most individual-level data, including residential census tract, smoking status, marital status, and income. In multiple-pollutant analyses, correlations among pollutants limit the ability to estimate independent associations between mortality risk and specific pollutants. For example, the correlation between PM_2.5_ and NO_2_ likely contributed to instability in the estimated effect of NO_2_ exposures; in models that controlled for PM_2.5_, NO_2_ was linked with decreased mortality risk. Similarly, in the temporally-decomposed analyses, the correlation of PM_2.5_ exposures over time made it difficult to determine the most relevant exposure window. In addition, the lack of variation in PM_2.5_-mortality associations between years may reflect a lack of independence between yearly cohorts, in which case the standard errors from fixed-effect meta-analytic estimates may be underestimated.

## Conclusions

Associations between long-term exposure to PM_2.5_ air pollution and mortality risk were robust to controlling for co-pollutants, observed across different spatial decompositions of PM_2.5_, and consistent over temporal decompositions of PM_2.5_. There was some evidence of increased toxicity for PM_2.5_ exposures that occurred closer to pollution sources. Exposures to SO_2_ and PM_2.5–10_ were also linked to mortality risk, even when controlling for other air pollutants.

## Additional file

**Additional file 1: Table S1.** Hazard ratios (and 95% CIs) from regressions using 6 criteria pollutants, scaled by IQR. **Table S2.** Hazard ratios (and 95% CIs) from spatially-decomposed analyses of PM_2.5._ **Table S3.** Hazard ratios (and 95% CIs) from temporally-decomposed PM_2.5_ and related analyses. **Figure S1.** Illustration of the construction of temporally decomposed cohorts
