# Supplementary material for: Air pollution and mortality in a large, representative U.S. cohort: multiple-pollutant analyses, and spatial and temporal decompositions
Source: Environ Health. 2019 Nov 21;18:101. doi: 10.1186/s12940-019-0544-9 (PMC6873509; doi:10.1186/s12940-019-0544-9)

**SUPPLEMENTARY MATERIAL**

Table S1. Hazard ratios (and 95% CIs) from regressions using 6 criteria pollutants, scaled by IQR.

|  | All-cause Mortality | Cardiopulmonary Mortality |
| --- | --- | --- |
| PM_2.5_ (IQR = 3.12 µg/m^3^) |  |  |
| alone | 1.038 (1.029 – 1.047) | 1.070 (1.055 – 1.085) |
| with PM_2.5-10_ | 1.036 (1.026 – 1.045) | 1.065 (1.050 – 1.080) |
| with SO_2_ | 1.035 (1.025 – 1.046) | 1.061 (1.044 – 1.077) |
| with NO_2_ | 1.050 (1.038 – 1.062) | 1.075 (1.056 – 1.094) |
| with O_3_ | 1.034 (1.024 – 1.044) | 1.058 (1.042 – 1.075) |
| with CO | 1.043 (1.032 – 1.053) | 1.069 (1.052 – 1.086) |
| with all pollutants (basic) | 1.045 (1.032 – 1.058) | 1.056 (1.035 – 1.077) |
| with all pollutants (complex) | 1.045 (1.030 – 1.061) | 1.057 (1.033 – 1.081) |
| PM_2.5-10_ (IQR = 5.43 µg/m^3^) |  |  |
| alone | 1.018 (1.008 – 1.028) | 1.038 (1.022 – 1.054) |
| with PM_2.5_ | 1.009 (0.999 – 1.019) | 1.022 (1.006 – 1.039) |
| with SO_2_ | 1.022 (1.012 – 1.032) | 1.047 (1.031 – 1.064) |
| with NO_2_ | 1.016 (1.005 – 1.026) | 1.026 (1.010 – 1.043) |
| with O_3_ | 1.015 (1.005 – 1.025) | 1.032 (1.016 – 1.048) |
| with CO | 1.016 (1.005 – 1.027) | 1.027 (1.009 – 1.044) |
| with all pollutants (basic) | 1.021 (1.009 – 1.033) | 1.034 (1.015 – 1.054) |
| with all pollutants (complex) | 1.025 (1.011 – 1.038) | 1.042 (1.020 – 1.063) |
| SO_2_ (IQR = 1.26 ppb) |  |  |
| alone | 1.023 (1.013 – 1.032) | 1.050 (1.035 – 1.065) |
| with PM_2.5_ | 1.006 (0.995 – 1.016) | 1.020 (1.004 – 1.037) |
| with PM_2.5-10_ | 1.026 (1.016 – 1.036) | 1.057 (1.041 – 1.073) |
| with NO_2_ | 1.022 (1.011 – 1.033) | 1.040 (1.023 – 1.057) |
| with O_3_ | 1.016 (1.006 – 1.026) | 1.035 (1.018 – 1.051) |
| with CO | 1.021 (1.011 – 1.032) | 1.042 (1.026 – 1.059) |
| with all pollutants (basic) | 1.019 (1.007 – 1.032) | 1.033 (1.014 – 1.053) |
| with all pollutants (complex) | 1.017 (1.003 – 1.030) | 1.027 (1.005 – 1.048) |
| NO_2_ (IQR = 6.72 ppb) |  |  |
| alone | 1.012 (1.002 – 1.022) | 1.041 (1.025 – 1.057) |
| with PM_2.5_ | 0.979 (0.968 – 0.991) | 0.992 (0.973 – 1.011) |
| with PM_2.5-10_ | 1.006 (0.996 – 1.017) | 1.031 (1.015 – 1.048) |
| with SO_2_ | 1.002 (0.992 – 1.013) | 1.023 (1.006 – 1.040) |
| with O_3_ | 1.008 (0.998 – 1.018) | 1.033 (1.017 – 1.049) |
| with CO | 1.008 (0.988 – 1.028) | 1.032 (1.001 – 1.065) |
| with all pollutants (basic) | 0.955 (0.933 – 0.977) | 0.950 (0.916 – 0.984) |
| with all pollutants (complex) | 0.956 (0.931 – 0.982) | 0.947 (0.912 – 0.983) |
| O_3_ (IQR = 6.75 ppb) |  |  |
| alone | 1.021 (1.013 – 1.030) | 1.047 (1.034 – 1.061) |
| with PM_2.5_ | 1.008 (0.999 – 1.017) | 1.024 (1.009 – 1.038) |
| with PM_2.5-10_ | 1.020 (1.012 – 1.028) | 1.044 (1.031 – 1.058) |
| with SO_2_ | 1.016 (1.008 – 1.025) | 1.036 (1.021 – 1.050) |
| with NO_2_ | 1.020 (1.012 – 1.029) | 1.043 (1.029 – 1.056) |
| with CO | 1.021 (1.013 – 1.030) | 1.047 (1.033 – 1.060) |
| with all pollutants (basic) | 1.002 (0.992 – 1.012) | 1.017 (1.001 – 1.033) |
| with all pollutants (complex) | 1.000 (0.989 – 1.011) | 1.016 (0.998 – 1.034) |
| CO (IQR = 99.08 ppb) |  |  |
| alone | 1.010 (1.002 – 1.019) | 1.033 (1.020 – 1.047) |
| with PM_2.5_ | 0.991 (0.982 – 1.001) | 1.002 (0.987 – 1.018) |
| with PM_2.5-10_ | 1.005 (0.995 – 1.014) | 1.024 (1.009 – 1.039) |
| with SO_2_ | 1.004 (0.995 – 1.013) | 1.021 (1.007 – 1.036) |
| with NO_2_ | 1.004 (0.987 – 1.022) | 1.008 (0.981 – 1.036) |
| with O_3_ | 1.010 (1.002 – 1.019) | 1.033 (1.019 – 1.047) |
| all pollutants (basic) | 1.013 (0.994 – 1.032) | 1.027 (0.997 – 1.057) |
| all pollutants (complex) | 1.008 (0.986 – 1.030) | 1.023 (0.993 – 1.054) |

Table S2. Hazard ratios (and 95% CIs) from spatially-decomposed analyses of PM_2.5_.

|  | All-cause Mortality | Cardiopulmonary Mortality |
| --- | --- | --- |
| Scaled by 10 µg/m^3^ |  |  |
| PM_2.5_, alone | 1.126 (1.095 – 1.158) | 1.172 (1.132 – 1.214) |
|  |  |  |
| Local PM_2.5_, alone | 1.563 (1.227 – 1.990) | 1.578 (1.079 – 2.306) |
| Neighborhood PM_2.5_, alone | 1.319 (1.211 – 1.436) | 1.517 (1.327 – 1.735) |
| Mid-range PM_2.5_, alone | 1.121 (1.066 – 1.179) | 1.249 (1.154 – 1.352) |
| Regional PM_2.5_, alone | 1.172 (1.109 – 1.238) | 1.377 (1.262 – 1.502) |
|  |  |  |
| Local PM_2.5_, joint | 1.299 (1.014 – 1.664) | 1.127 (0.764 – 1.662) |
| Neighborhood PM_2.5_, joint | 1.279 (1.173 – 1.395) | 1.451 (1.266 – 1.663) |
| Mid-range PM_2.5_, joint | 1.094 (1.038 – 1.153) | 1.183 (1.089 – 1.285) |
| Regional PM_2.5_, joint | 1.106 (1.043 – 1.173) | 1.264 (1.153 – 1.386) |
|  |  |  |
| Scaled by IQR |  |  |
| PM_2.5_, alone | 1.038 (1.029 – 1.047) | 1.070 (1.055 – 1.085) |
|  |  |  |
| Local PM_2.5_, alone | 1.014 (1.007 – 1.022) | 1.015 (1.002 – 1.027) |
| Neighborhood PM_2.5_, alone | 1.029 (1.020 – 1.037) | 1.043 (1.029 – 1.058) |
| Mid-range PM_2.5_, alone | 1.018 (1.010 – 1.026) | 1.035 (1.022 – 1.047) |
| Regional PM_2.5_, alone | 1.043 (1.028 – 1.058) | 1.089 (1.064 – 1.114) |
|  |  |  |
| Local PM_2.5_, joint | 1.008 (1.000 – 1.017) | 1.004 (0.991 – 1.016) |
| Neighborhood PM_2.5_, joint | 1.025 (1.016 – 1.034) | 1.039 (1.024 – 1.053) |
| Mid-range PM_2.5_, joint | 1.014 (1.006 – 1.022) | 1.026 (1.013 – 1.039) |
| Regional PM_2.5_, joint | 1.027 (1.011 – 1.043) | 1.064 (1.038 – 1.090) |

Table S3. Hazard ratios (and 95% CIs) from temporally-decomposed PM_2.5_ and related analyses.

|  | All-cause Mortality | Cardiopulmonary Mortality |
| --- | --- | --- |
| PM_2.5_, 1988-2015, basic model | 1.090 (1.066 – 1.115) | 1.172 (1.132 – 1.214) |
| PM_2.5_, 1999-2015, complex model | 1.119 (1.085 – 1.155) | 1.227 (1.166 – 1.290) |
|  |  |  |
| PM_2.5_, 5-yr mean, fixed effect | 1.095 (1.068 – 1.124) | 1.168 (1.125 – 1.212) |
| PM_2.5_, 2-yr mean, fixed effect | 1.101 (1.073 – 1.129) | 1.181 (1.135 – 1.228) |
|  |  |  |
| PM_2.5_, 2-yr mean, 1992 cohort | 1.077 (0.925 – 1.256) | 1.161 (0.946 – 1.424) |
| PM_2.5_, 2-yr mean, 1993 cohort | 1.040 (0.906 – 1.193) | 1.190 (0.981 – 1.442) |
| PM_2.5_, 2-yr mean, 1994 cohort | 1.146 (1.027 – 1.278) | 1.355 (1.155 – 1.589) |
| PM_2.5_, 2-yr mean, 1995 cohort | 1.058 (0.953 – 1.175) | 0.996 (0.854 – 1.162) |
| PM_2.5_, 2-yr mean, 1996 cohort | 1.143 (1.031 – 1.268) | 1.083 (0.931 – 1.258) |
| PM_2.5_, 2-yr mean, 1997 cohort | 1.049 (0.926 – 1.189) | 0.957 (0.801 – 1.143) |
| PM_2.5_, 2-yr mean, 1998 cohort | 1.070 (0.933 – 1.226) | 1.388 (1.130 – 1.707) |
| PM_2.5_, 2-yr mean, 1999 cohort | 1.128 (1.016 – 1.252) | 1.498 (1.276 – 1.758) |
| PM_2.5_, 2-yr mean, 2000 cohort | 1.097 (0.976 – 1.234) | 1.216 (1.029 – 1.436) |
| PM_2.5_, 2-yr mean, 2001 cohort | 0.986 (0.883 – 1.101) | 1.035 (0.878 – 1.220) |
| PM_2.5_, 2-yr mean, 2002 cohort | 0.990 (0.887 – 1.105) | 1.115 (0.946 – 1.314) |
| PM_2.5_, 2-yr mean, 2003 cohort | 1.018 (0.908 – 1.142) | 1.104 (0.948 – 1.284) |
| PM_2.5_, 2-yr mean, 2004 cohort | 1.171 (1.042 – 1.317) | 1.141 (0.947 – 1.376) |
| PM_2.5_, 2-yr mean, 2005 cohort | 1.100 (0.973 – 1.243) | 1.185 (0.955 – 1.470) |
| PM_2.5_, 2-yr mean, 2006 cohort | 1.160 (1.011 – 1.332) | 1.336 (1.089 – 1.639) |
| PM_2.5_, 2-yr mean, 2007 cohort | 1.033 (0.904 – 1.181) | 0.997 (0.795 – 1.250) |
| PM_2.5_, 2-yr mean, 2008 cohort | 1.348 (1.182 – 1.537) | 1.489 (1.198 – 1.850) |
| PM_2.5_, 2-yr mean, 2009 cohort | 1.142 (0.986 – 1.324) | 1.372 (1.082 – 1.739) |
| PM_2.5_, 2-yr mean, 2010 cohort | 1.070 (0.910 – 1.258) | 1.098 (0.845 – 1.426) |
| PM_2.5_, 2-yr mean, 2011 cohort | 1.251 (1.058 – 1.480) | 1.267 (0.971 – 1.654) |
| PM_2.5_, 2-yr mean, 2012 cohort | 1.229 (1.023 – 1.478) | 1.253 (0.924 – 1.698) |
| PM_2.5_, 2-yr mean, 2013 cohort | 1.135 (0.947 – 1.361) | 1.253 (0.909 – 1.729) |
| PM_2.5_, 2-yr mean, 2014 cohort | 1.168 (0.984 – 1.385) | 1.284 (0.940 – 1.754) |
| PM_2.5_, 2-yr mean, 2015 cohort | 1.102 (0.936 – 1.298) | 1.176 (0.894 – 1.546) |

Figure S1. Illustration of the construction of temporally decomposed cohorts.


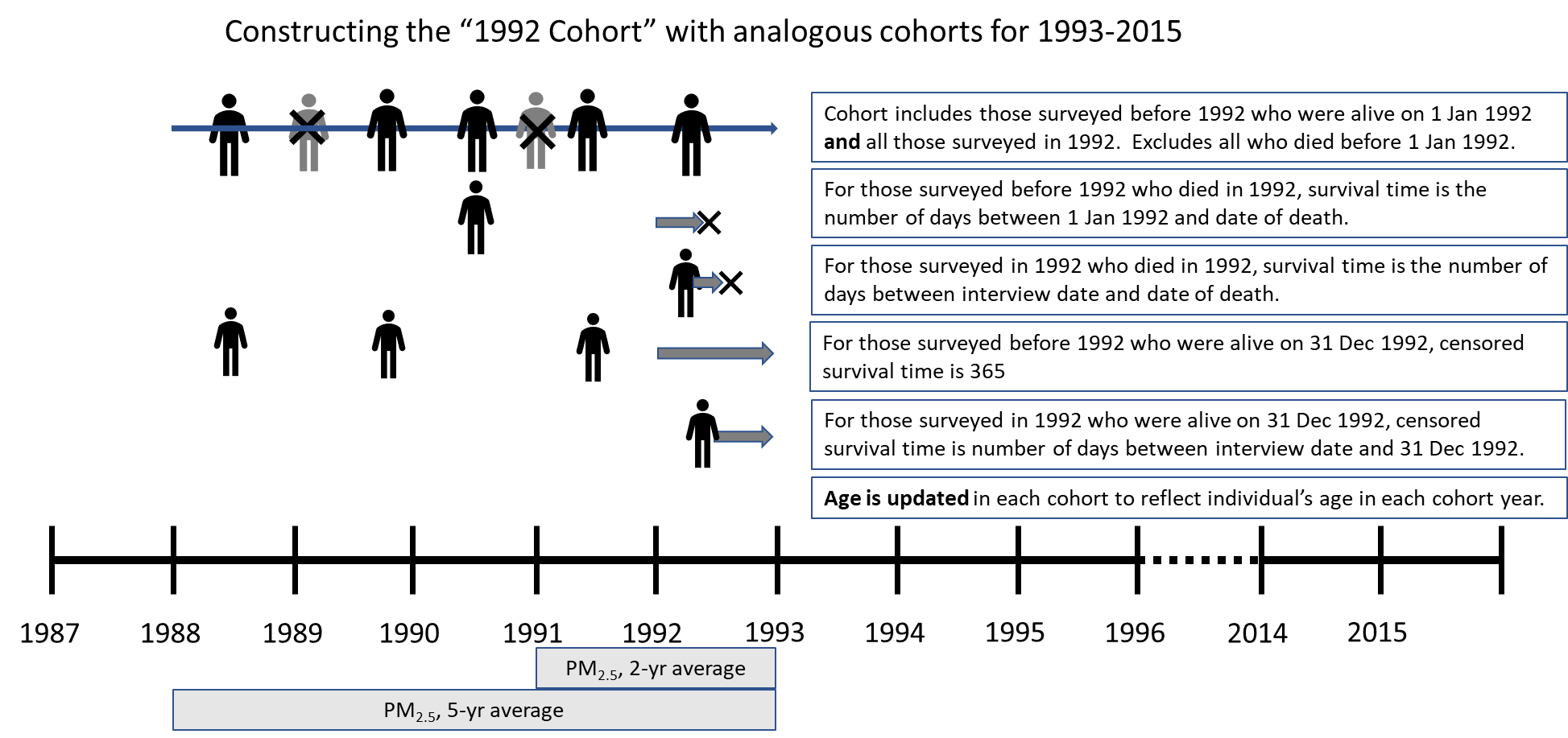

Supplement: Supplementary file 1 — Additional file 1: Table S1. Hazard ratios (and 95% CIs) from regressions using 6 criteria pollutants, scaled by IQR. Table S2. Hazard ratios (and 95% CIs) from spatially-decomposed analyses of PM2.5. Table S3. Hazard ratios (and 95% CIs) from temporally-decomposed PM2.5 and related analyses. Figure S1. Illustration of the construction of temporally decomposed cohorts [file 12940_2019_544_MOESM1_ESM.docx]
